# Development and Validation of a Computed Tomography–Based Model for Noninvasive Prediction of the T Stage in Gastric Cancer: Multicenter Retrospective Study

**DOI:** 10.2196/56851

**Published:** 2024-10-09

**Authors:** Jin Tao, Dan Liu, Fu-Bi Hu, Xiao Zhang, Hongkun Yin, Huiling Zhang, Kai Zhang, Zixing Huang, Kun Yang

**Affiliations:** 1 Department of General Surgery and Laboratory of Gastric Cancer State Key Laboratory of Biotherapy/Collaborative Innovation Center of Biotherapy and Cancer Center West China Hospital, Sichuan University Chengdu China; 2 Gastric Cancer Center West China Hospital Sichuan University Chengdu China; 3 Department of Radiology West China Hospital Sichuan University Chengdu China; 4 Department of Radiology The First Affiliated Hospital of Chengdu Medical College Chengdu China; 5 Department of Radiology People’s Hospital of Leshan Leshan China; 6 Institute of Advanced Research Infervision Medical Technology Co Ltd Beijing China

**Keywords:** gastric cancer, computed tomography, radiomics, T stage, deep learning, cancer, multicenter study, accuracy, binary classification, tumor, hybrid model, performance, pathological stage

## Abstract

**Background:**

As part of the TNM (tumor-node-metastasis) staging system, T staging based on tumor depth is crucial for developing treatment plans. Previous studies have constructed a deep learning model based on computed tomographic (CT) radiomic signatures to predict the number of lymph node metastases and survival in patients with resected gastric cancer (GC). However, few studies have reported the combination of deep learning and radiomics in predicting T staging in GC.

**Objective:**

This study aimed to develop a CT-based model for automatic prediction of the T stage of GC via radiomics and deep learning.

**Methods:**

A total of 771 GC patients from 3 centers were retrospectively enrolled and divided into training, validation, and testing cohorts. Patients with GC were classified into mild (stage T1 and T2), moderate (stage T3), and severe (stage T4) groups. Three predictive models based on the labeled CT images were constructed using the radiomics features (radiomics model), deep features (deep learning model), and a combination of both (hybrid model).

**Results:**

The overall classification accuracy of the radiomics model was 64.3% in the internal testing data set. The deep learning model and hybrid model showed better performance than the radiomics model, with overall classification accuracies of 75.7% (*P*=.04) and 81.4% (*P*=.001), respectively. On the subtasks of binary classification of tumor severity, the areas under the curve of the radiomics, deep learning, and hybrid models were 0.875, 0.866, and 0.886 in the internal testing data set and 0.820, 0.818, and 0.972 in the external testing data set, respectively, for differentiating mild (stage T1~T2) from nonmild (stage T3~T4) patients, and were 0.815, 0.892, and 0.894 in the internal testing data set and 0.685, 0.808, and 0.897 in the external testing data set, respectively, for differentiating nonsevere (stage T1~T3) from severe (stage T4) patients.

**Conclusions:**

The hybrid model integrating radiomics features and deep features showed favorable performance in diagnosing the pathological stage of GC.

## Introduction

Gastric cancer (GC) is one of the most prevalent cancers and ranks as the fourth most common cause of cancer-related death globally [1]. Although systemic therapy can increase the survival rate and improve quality of life, the prognosis remains poor due to diagnosis at an advanced stage [2]. Currently, GC staging is performed according to the Union for International Cancer Control and American Joint Committee on Cancer tumor‐node‐metastasis (TNM) staging system [3]. This system could be used to stratify cancer prognosis. As part of the TNM staging system, T staging based on the tumor depth of GC is crucial for developing treatment plans. For patients with clinical stage T1, endoscopic procedures have been considered the first choice, while for those with clinical stages T2, T3, or T4, surgery and perioperative chemotherapy have been recommended [4]. However, the clinical and pathological diagnosis of tumor depths can sometimes vary [5]. In preoperative T staging, computed tomography (CT) has a sensitivity and specificity of 80% to 90%, respectively, for discriminating between early- and advanced-stage GCs [6], and precise prediction of preoperative T staging using CT plays a vital role in GC treatment.

A retrospective study [7] involving 244 patients with GC reported that the performance of single-phase CT radiomics models is favorable in differentiating between T2- and T3/T4-stage tumors [7]. By extracting intricate information that is imperceptible to the human eye in medical imaging and transforming it into quantitative data, radiomics and deep learning approaches have shown potential in improving the diagnostic capability of current imaging. In radiomics, medical images are transformed into mineable high-dimensional data, which can be used to quantify lesion heterogeneity that cannot be observed in the images. Furthermore, deep learning is the state-of-the-art machine learning approach that uses multiple processing layers and connections to learn complex relationships between input data and desired outputs from a large number of labeled examples, which could provide clinicians with decision support and improve diagnostic and treatment accuracy and efficiency [8,9]. Previous studies have constructed a deep learning model based on CT imaging radiomics signatures to predict the number of lymph node metastases and survival in patients with resected GC [10,11]. Our team has previously studied occult peritoneal metastasis of GC by using imaging radiomics and deep learning [12,13]. In addition, the feasibility and promising performance of machine learning approaches in assessing T staging in lung cancer has been demonstrated [14]. However, few studies have reported the combination of deep learning and radiomics in predicting T staging in GC.

To improve the diagnostic accuracy of T staging to better develop treatment strategies, this study focused on preoperative differentiation among pathological T stages using deep learning and combining single-phase CT radiomics models with deep learning parameters, which can potentially be helpful and assist in guiding clinicians in regard to personalized medicine.

## Methods

### Patient Enrollment and Eligibility Criteria

The inclusion criteria were as follows: (1) primary gastric adenocarcinoma diagnosed via endoscopy-biopsy pathology; (2) venous images of the whole abdomen (with a slice thickness of 2 mm) obtained preoperatively followed by laparoscopy or surgery performed within 2 weeks; (3) no typical peritoneal metastasis findings, such as omental nodules or omental cake, extensive ascites, or irregular thickening with high peritoneal enhancement, on CT; and (4) no indications of distant metastasis or other tumors.

The exclusion criteria were as follows: (1) previous abdominal surgery, (2) previous abdominal malignancies or inflammatory diseases, (3) insufficient distention of the stomach, (4) poor imaging quality due to artefacts, and (5) indiscernible primary GC tumor on CT images.

### Ethical Considerations

This study was granted approval by the ethics committee of West China Hospital, Sichuan University (2019-1158). Given the retrospective nature of the study and the anonymous analysis of all data, the need for informed consent was waived.

### Segmentation and Preprocessing of Tumor Regions

Given the T stage in GC, we divided the images into 3 gradings for this study: mild (stages T1 and T2), moderate (stage T3), and severe (stages T4a and T4b) groups. The open-source software ITK-SNAP (version 3.8.0; University of North Carolina) was used for image segmentation. The outline for the tumor regions of interest (ROIs) was manually drawn by a junior radiologist with 6 years of experience in reading abdominal CT images and revised by a senior radiologist with 17 years of experience. When delineating the tumor ROIs, the radiologist referred to the results of gastroscopy to determine the location of the tumor. Besides, 40 CT images of GC lesions were randomly selected and delineated again by the junior radiologist to assess test-retest reliability. Blinded to segmentations delineated by the junior radiologist, these 40 CT images of GC lesions were delineated again by the senior radiologist to assess the intraclass correlation coefficient (ICC). Features with an interobserver ICC of >0.85 were retained, and the ICC values of the selected radiomics features are presented in Table S2 in Multimedia Appendix 1.

### Extraction and Selection of the Radiomics Features

The radiomics features were automatically extracted from the ROIs on the maximum cross-section layer of the noncontrast CT images by using the PyRadiomics package (version 3.0). A set of filters (Wavelet, Square, SquareRoot, and Logarithm) were applied to highlight certain image properties [15]. Finally, a total of 1183 radiomics features were extracted from each of the patients. Principal component analysis was used for dimension reduction of the features, to reduce computation complexity, and to prevent overfitting. Principal component analysis is an unsupervised method that transforms complex, high-dimensional original radiomics data into a dimensionally reduced set of uncorrelated features named “principal components.” Principal components were computed based on singular value decomposition of the standardized radiomics features, and the top 15 components that explained most of the variance in the training data set were finally selected for model development.

### CT Image Acquisition

**Figure 1:**
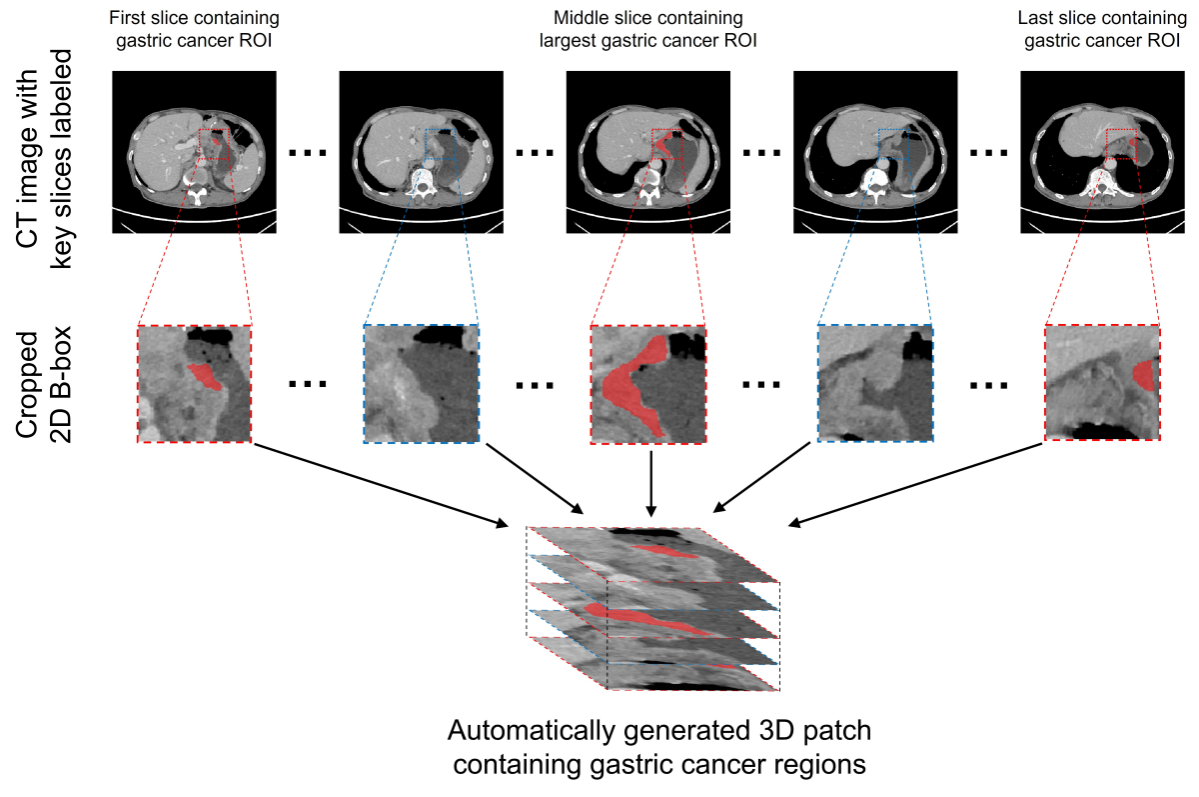
Example of the key slice–based automatic generation of a 3D cube including the whole gastric cancer region. CT: computed tomography; ROI: region of interest.

The details of the CT protocol are presented in Section 2 and Table S1 in the Multimedia Appendix 1. Segmentation and preprocessing of tumor ROIs, Extraction and selection of the radiomics features are shown in Multimedia Appendix 1. To reduce the radiologists’ workload of depicting the ROIs, this study attempted to apply partial markers to automatically generate 3D patches. The radiologists manually delineated the ROIs in the initial image, the final image, and maximum cross-section layers with the largest tumor boundaries. Based on information regarding the ROIs of the 3 key layers, 3D cubes including the whole tumor and the surrounding structures were automatically generated (Figure 1).

### Development of the Radiomics Model

Based on the selected radiomics features, a radiomics model was constructed by using the support vector machine classifier. As a supervised learning method that was very effective in linear or nonlinear classification tasks, the support vector machine classifier has been widely used in radiomics analysis. The radiomics model was developed and validated on the InferScholar (version 3.5) platform, and the parameters were set as follows: C=1.0, kernel=“Sigmoid,” gamma=“auto,” Tol=0.001, class_weight=“balanced,” and other parameters were set as default.

### Development of the Deep Learning Models

#### Vision Transformer–Based Deep Learning Model

**Figure 2:**
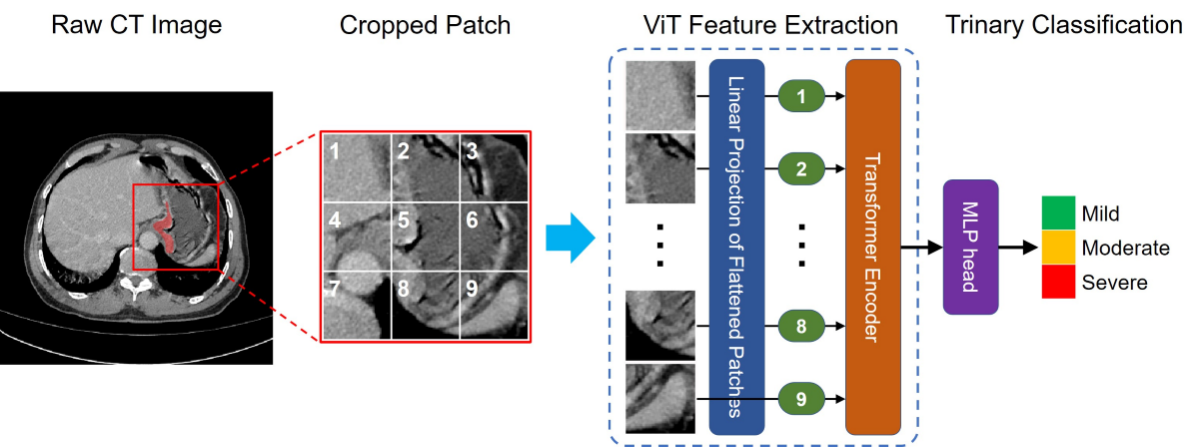
Overview of the vision transformer (ViT) for the pathological severity classification of gastric cancer on noncontrast computed tomography (CT) images.

Vision transformers (ViTs), derived by pure transformers, were first proposed for natural language processing by Vaswani et al [16]. In this study, we applied a ViT to grade GC severity based on CT images. The current implementation is inspired by the work of Alexey Dosovitskiy [17] and his team, who applied ViT to the ImageNet data set and showed excellent performance in image classification compared with state-of-the-art convolutional neural networks. Due to its advantage of analyzing overall relationships by modeling the interrelationships among different parts of an image, ViT showed strength in global image classification, which is very suitable for our case [18]. The conceptual architecture of the ViT used in our study is illustrated in Figure 2. Briefly, the 3D patches containing GC regions were cropped from CT images. After resizing, the 3D patch was split into small patches, which, in turn, were converted to a sequence of patch embeddings via flattening and linear projection. Then, the patch embeddings and the positional embedding were fed into the transformer encoder to obtain the final representation. Consequently, the learnable features in the inputted images were fed into the classifier head to identify GC severity. In this study, the embedding dimension of the ViT models was set at 768, the numbers of both encoder layers and the attention heads were 12, and the dimensionality of the expanded representation in the predicting head was 3072.

#### Deep Learning–Based Hybrid Model

To integrate radiomics features and deep learning features, a ViT architecture–based hybrid model was proposed in this study, namely, the ViT-Radiomics model [19,20], which combined both ViT and radiomics models for grading GC severity. In brief, the deep features extracted by the ViT were first transformed into a 1280-bit vector, while the selected radiomics features were also transformed into a 512-bit vector by using a vectorization transform approach. Then, the vectorized deep features and radiomics features were further concatenated into a 1792-bit vector and used to predict the severity of gastric cancer (mild, moderate, or severe).

### Data Augmentation

To meet the high demand for large amounts of training data in ViT-based deep learning models, several sophisticated data augmentation techniques were applied in the training data set to avoid the overfitting problem and promote prediction performance during the model training process. Data augmentation techniques in this study mainly referred to a series of geometric transformations including scaling (0.9 times or 1.1 times the image size), translation (up and down, left and right, and front and back), mirroring, rotation, and flip (horizontal and vertical) on the generated 3D patches of the original images. After data augmentation, the sample size in the training set increased to 5 times that of the original training data set.

### Training of the Deep Learning Models

The deep learning models (deep learning and hybrid models) were trained by maximizing their identification performance (ie, accuracy) for grading GC severity and minimizing the categorical cross-entropy loss. This study used stochastic gradient descent with a momentum of 0.95, a weight decay of 0.0001, and an initial learning rate of 0.001 to optimize the model’s parameters. The number of modeling epochs was set to 150, and the minibatch size was set to 32. The model development process was implemented using 4 GeForce RTX 2080ti GPUs on Ubuntu 18.04.4 LTS, Python 3.7.11, and PyTorch 1.7.1. No samples overlapped at the patient level in the development and independent data sets.

### Evaluation of Model Performance

The trinary diagnostic capability of the predictive models was assessed on the basis of overall accuracy and the Cohen κ coefficient [21], and the chi-square test was used to compare the accuracies of different predictive models. Because of the imbalanced distribution of the 3 GC severity categories, the per-class *F*_1_-score and weighted average *F*_1_-score were also calculated [22].

In addition, two clinically important subtasks were also evaluated: (1) binary classification of mild (stages T1~T2) GCs and nonmild GCs (stages T3~T4) and (2) binary classification of nonsevere GCs (stages T1~T3) and severe GCs (stage T4). The discriminative efficacy of binary classification was evaluated using receiver operating characteristic analysis using the area under the curve (AUC). The sensitivity, specificity, positive predictive value, and negative predictive value were also calculated under the optimal threshold in accordance with the maximum Youden index [23]. Furthermore, decision curve analysis was used to evaluate the clinical utility of the radiomics, deep learning, and hybrid models for binary classification of mild/nonmild and nonsevere/severe GCs by comparing the net benefit across a range of threshold probabilities in the training and validation data sets [24].

### Statistical Analysis

Statistical analysis was performed using R (version 3.3.1; The R Foundation), SPSS (version 23.0; IBM Corp), and MedCalc (version 20.0; MedCalc). The differences in continuous variables with normal or nonnormal distributions were evaluated using a 2-tailed Student *t* test or the Mann-Whitney *U* test, respectively. Categorical variables were compared using the chi-square test. Differences between the 2 AUCs of different models were assessed by using the DeLong test [25]. A decision curve was plotted using the “rmda” package in R. A 2-sided *P* value less than .05 was considered statistically significant.

## Results

### Patient Characteristics

Ultimately, a total of 706 patients with GC were enrolled in this retrospective study and randomized 4:1 into a development data set (n=566) and an independent testing data set (n=140). In total, 65 patients with GC from 2 external centers were also recruited as the external testing data set (Figure S1 in Multimedia Appendix 1).

Clinical characteristics in the development, internal, and external sets are listed in Table 1. Age, peritoneum metastasis, and carbohydrate antigen 19-9 (CA19-9) differed significantly among the 3 data sets. No significant difference was observed among the 3 data sets in terms of sex, size, site, Boorman type, grade, Lauren type, adjacent tissue invasion, pathological T stage, TNM stage, serum cancer antigen 72-4 (CA72-4) levels, serum cancer antigen 125 (CA125) levels, and carcinoembryonic antigen (CEA) levels.

**Table 1:** Characteristics of the enrolled patients with gastric cancer in the development, internal, and external data sets.

Variables		Training (n=566)	Testing (n=140)	External (n=65)	*P* value	
**Sex, n (%)**					.85	
	Male	372 (65.72)	93 (66.43)	45 (69.23)		
	Female	194 (34.28)	47 (33.57)	20 (30.77)		
Age (years), median (IQR)		60 (51-67)	58 (49-65)	66 (57-70)	.001	
Tumor size (cm), median (IQR)		5 (3-7)	5 (3-7)	5 (3-6)	.69	
**Tumor location, n (%)**						.30
	U	93 (16.43)	19 (13.57)	9 (13.85)		
	M	73 (12.90)	16 (11.43)	7 (10.77)		
	L	300 (53.00)	74 (52.86)	35 (53.85)		
	U+E	7 (1.24)	9 (6.43)	3 (4.62)		
	UM/MU	17 (3.00)	6 (4.29)	3 (4.62)		
	LM/ML	45 (7.95)	11 (7.86)	4 (6.15)		
	L+D	7 (1.24)	1 (0.71)	0 (0)		
	LMU/MLU/MUL/UML	24 (4.24)	4 (2.86)	4 (6.15)		
**Peritoneal metastasis, n (%)**						.03
	Negative	363 (64.13)	95 (67.86)	52 (80.00)		
	Positive	203 (35.87)	45 (32.14)	13 (20.00)		
**Borrmann classification, n (%)**						.11
	Early stage	94 (16.61)	22 (15.71)	1 (1.54)		
	I	19 (3.36)	6 (4.29)	3 (4.62)		
	II	157 (27.74)	45 (32.14)	18 (27.69)		
	III	235 (41.52)	52 (37.14)	34 (52.31)		
	IV	61 (10.78)	15 (10.71)	9 (13.85)		
**Grade, n (%)**						.14
	Unknown	14 (2.48)	6 (4.29)	3 (4.62)		
	1	217 (38.34)	54 (38.57)	20 (30.8)		
	2	325 (57.42)	78 (55.71)	37 (56.92)		
	3	1 (0.18)	0 (0)	0 (0)		
	4	9 (1.59)	2 (1.43)	5 (7.7)		
**Lauren histological type, n (%)**						.67
	Intestinal	170 (30.04)	40 (28.57)	15 (23.08)		
	Diffuse	249 (43.99)	62 (44.29)	28 (43.08)		
	Mixed	147 (25.97)	38 (27.14)	22 (33.85)		
**Adjacent tissues invaded by tumors, n (%)**						.28
	Negative	502 (88.69)	127 (90.71)	54 (83.08)		
	Positive	64 (11.31)	13 (9.29)	11 (16.92)		
**T stage, n (%)**						.22
	T1a	57 (10.07)	14 (10.00)	2 (3.08)		
	T1b	57 (10.07)	15 (10.71)	3 (4.62)		
	T2	102 (18.02)	24 (17.14)	8 (12.30)		
	T3	108 (19.08)	27 (19.29)	11 (16.92)		
	T4a	187 (33.04)	49 (35.00)	34 (52.31)		
	T4b	55 (9.72)	11 (7.86)	7 (10.77)		
**TNM^a^** **stage, n (%)**						.88
	IA	33 (5.83)	9 (6.43)	5 (7.69)		
	IB	59 (10.42)	15 (10.71)	7 (10.77)		
	IIA	77 (13.60)	12 (8.57)	5 (7.69)		
	IIB	75 (13.25)	25 (17.86)	8 (12.31)		
	IIIA	56 (9.89)	11 (7.86)	7 (10.77)		
	IIIB	80 (14.13)	20 (14.29)	8 (12.31)		
	IIIC	94 (16.61)	24 (17.14)	10 (15.38)		
	IV	92 (16.25)	24 (17.14)	15 (23.08)		
Serum cancer antigen 72-4 level (ng/mL), median (IQR)		3.55 (1.78-8.58)	3.46 (1.89-7.59)	3.04 (1.20-10.55)	.70	
Carbohydrate antigen 19-9 level (kU/L), median (IQR)		10.50 (6.44-20.71)	9.50 (5.88-18.09)	13.20 (8.45-27.20)	.03	
Serum cancer antigen level (U/mL), median (IQR)		11.50 (8.65-17.23)	12.12 (8.16-19.48)	10.00 (7.13-13.82)	.07	
Carcinoembryonic antigen level (ng/mL), median (IQR)		2.00 (1.32-3.28)	2.19 (1.40-4.15)	1.77 (1.06-4.15)	.45	

^a^TNM: tumor‐node‐metastasis.

### Performance Evaluation of the Trinary Classification of GC Severity

**Figure 3:**
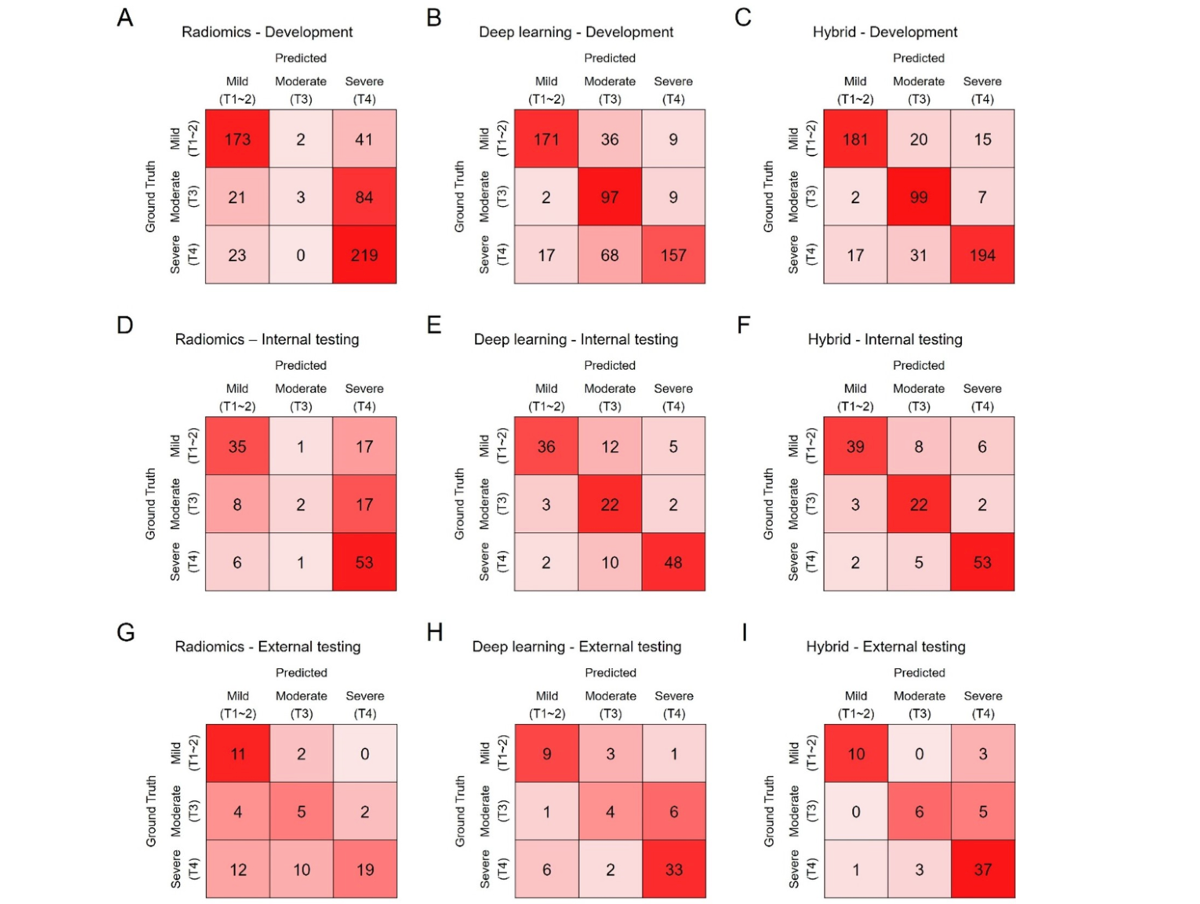
Trinary classification performance of the predictive models. (A)-(C) The 3-class confusion matrix of the radiomics, deep learning, and hybrid models in the development data set. (D)-(F) The 3-class confusion matrix of the radiomics, deep learning, and hybrid models in the internal testing data set. (G)-(I) The 3-class confusion matrix of the radiomics, deep learning, and hybrid models in the external testing data set.

The 3-class confusion matrices of the predictive models in the development, internal testing, and external testing data sets are shown in Figure 3. The overall accuracies of the radiomics, deep learning, and hybrid models were 69.8%, 75.1%, and 83.7% in the development data set; 64.3%, 75.7%, and 81.4% in the internal testing data set; and 53.8%, 70.8%, and 81.5% in the external testing data set, respectively. The accuracy of the deep learning and hybrid models were significantly different from those of the radiomics model in the development testing (deep learning vs radiomics: *P*=.046; hybrid vs radiomics: *P*<.001), internal testing (deep learning vs radiomics: *P*=.04; hybrid vs radiomics: *P*=.001), and external testing (deep learning vs radiomics: *P*=.047; hybrid vs radiomics: *P*<.001) data sets. The overall accuracy of the hybrid model was significantly different from that of the deep learning model in the development data set (*P*<.001); however, no significant difference was observed in the internal (*P*=.25) or external (*P*=.15) testing data sets. Similarly, the predicted results of both the deep learning and hybrid models showed high agreement with the pathologically confirmed ground truth in the development (Cohen κ=0.660 for the deep learning model and 0.767 for the hybrid model), internal testing (Cohen κ=0.665 for the deep learning model and 0.735 for the hybrid model), and external testing (Cohen κ=0.689 for the hybrid model) data sets, while the agreement for the radiomics model was only moderate (Cohen κ=0.579 for the development, 0.518 for the internal testing, and 0.400 for the external testing data sets). These results, as well as the per-class *F*_1_-score and weighted average *F*_1_-score, are presented in Table 2.

**Table 2:** Trinary classification performance of the models in predicting the T stage of gastric cancer.

Data set and model		Accuracy, %		*F*_1_-score (mild)		*F*_1_-score (moderate)		*F*_1_-score (severe)		*F*_1_-score (average)		Cohen κ
**Development data set**												
	Radiomics	69.8	0.799		0.053		0.747		0.635		0.579	
	Deep learning	75.1	0.842		0.628		0.753		0.763		0.660	
	Hybrid	83.7	0.870		0.767		0.847		0.841		0.767	
**Internal testing data set**												
	Radiomics	64.3	0.686		0.129		0.721		0.594		0.518	
	Deep learning	75.7	0.766		0.620		0.835		0.767		0.665	
	Hybrid	81.4	0.804		0.710		0.876		0.817		0.735	
**External testing data set**												
	Radiomics	53.8	0.550		0.357		0.613		0.557		0.400	
	Deep learning	70.8	0.621		0.400		0.815		0.706		0.544	
	Hybrid	81.5	0.833		0.600		0.860		0.811		0.689	

### Evaluation on 2 Binary Classification Subtasks

**Figure 4:**
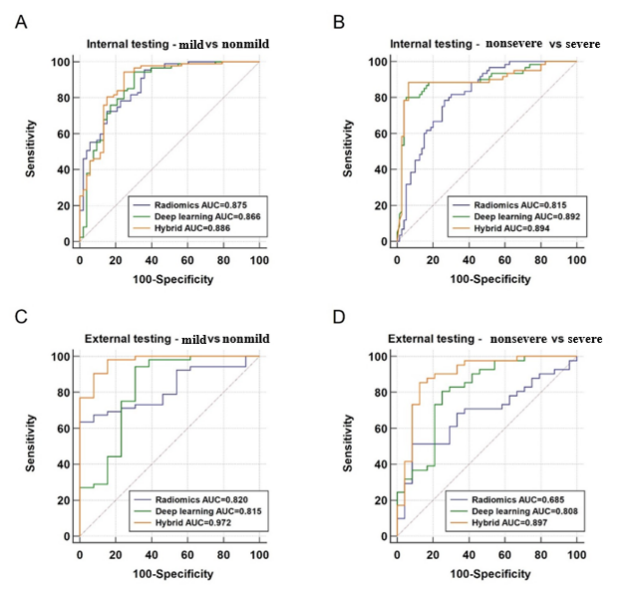
Comparison between the predictive models on 2 binary subtasks. AUC: area under the curve.

As depicted in Figure 4, the binary classification performance of the predictive models for mild versus nonmild and nonsevere versus severe GCs was evaluated in both internal and external testing data sets. For binary classification of mild (stage T1~T2) and nonmild (stage T3~T4) GCs, the radiomics, deep learning, and hybrid models showed similar performance, with AUCs of 0.875 (95% CI 0.809-0.925), 0.866 (95% CI 0.799-0.918), and 0.886 (95% CI 0.822-0.934) in the internal testing data set, respectively (Delong test; *P*>.05 for all). Meanwhile, The AUC values of the hybrid, radiomics, and deep learning models in the external testing data set were 0.972 (95% CI 0.897-0.997), 0.820 (95% CI 0.704-0.904; *P*=.002), and 0.818 (95% CI 0.703-0.903; *P*=.03), respectively. For binary classification of nonsevere (stage T1~T3) and severe (stage T4) GCs, the AUC values of deep learning and hybrid models were 0.892 (95% CI 0.829-0.938) and 0.894 (95% CI 0.831-0.940), respectively. Furthermore, the AUC value of the radiomics model was 0.815 (95% CI 0.740-0.875; radiomics vs deep learning: *P*=.02; radiomics vs hybrid: *P*=.03). In addition, the AUC values for the deep learning, hybrid, and radiomics models were 0.808 (95% CI 0.691-0.895), 0.897 (95% CI 0.797-0.959), and 0.685 (95% CI 0.558-0.795), respectively (radiomics vs deep learning: *P*=.03; radiomics vs hybrid: *P*=.002), in the external testing data set, and no significant difference was observed between them (*P*=.10). The detailed performances of these models in the internal and external testing data sets are listed in Tables 3 and 4, respectively.

**Table 3:** Comparison of the performance of the predictive models for binary classification of mild and nonmild gastric cancers in the internal and external testing data sets.

Data set and model		Area under the curve (95% CI)	Sensitivity, %	Specificity, %	Positive predictive value, %	Negative predictive value, %
**Internal testing data set**						
	Radiomics	0.875 (0.809-0.925)	95.4	64.2	81.4	89.5
	Deep learning	0.866 (0.799-0.918)	94.3	69.8	83.7	88.1
	Hybrid	0.886 (0.822-0.934)	94.3	75.5	86.3	88.9
**External testing data set**						
	Radiomics	0.820 (0.704-0.904)	63.5	100	100	40.6
	Deep learning	0.818 (0.703-0.903)	94.2	69.2	92.5	75.0
	Hybrid	0.972 (0.897-0.997)	90.4	92.3	97.9	70.6

**Table 4:** Comparison of the performance of the predictive models for binary classification of nonsevere and severe gastric cancers in the internal and external testing data sets.

Data set and model		Area under the curve (95% CI)	Sensitivity, %	Specificity, %	Positive predictive value, %	Negative predictive value, %
**Internal testing data set**						
	Radiomics	0.815 (0.740-0.875)	78.3	73.8	69.1	81.9
	Deep learning	0.892 (0.829-0.938)	80.0	95.0	92.3	86.4
	Hybrid	0.894 (0.831-0.940)	88.3	93.8	91.4	91.5
**External testing data set**						
	Radiomics	0.685 (0.558-0.795)	51.2	91.7	91.3	52.4
	Deep learning	0.808 (0.691-0.895)	80.5	75.0	84.6	69.2
	Hybrid	0.897 (0.797-0.959)	85.4	87.5	92.1	77.8

### Clinical Utility Analysis

**Figure 5:**
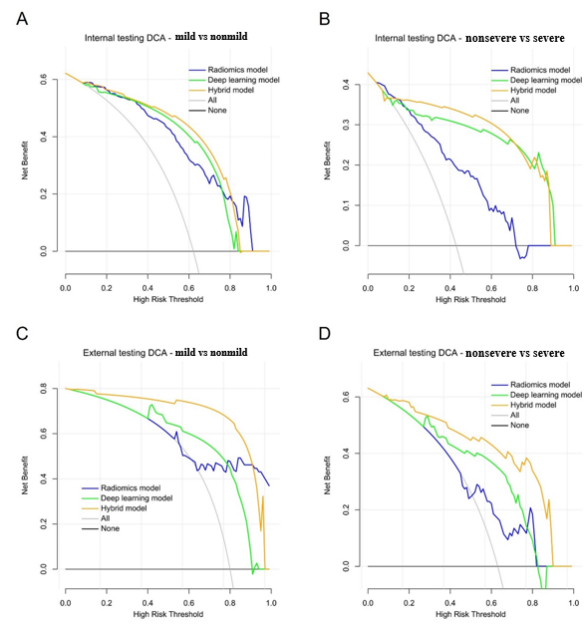
Decision curve analysis (DCA) for the predictive models in the internal testing data set on (A) the binary classification subtask 1 (stage T1~T2 vs stage T3~T4) and (B) the binary classification subtask 2 (stage T1~T3 vs stage T4) and for the predictive models on binary classification subtask 1 (C) and 2 (D) in the external testing data set. The net benefit is depicted on the y-axis. The gray line and black line represent situations in which all patients and no patients underwent biopsy/surgery, respectively.

Decision curve analyses for the 2 binary classification subtasks of the different predictive models in the internal and external data sets are presented in Figure 5. During binary classification of mild (stage T1~T2) and nonmild (stage T3~T4) GCs, the hybrid model had a slightly higher overall net benefit than the radiomics and deep learning models across the majority range of reasonable threshold probabilities in the internal testing data set. For binary classification of nonsevere (stage T1~T3) and severe (stage T4) GCs, the deep learning and hybrid models showed a markedly higher net benefit than the radiomics model across the majority range of reasonable threshold probabilities in the internal testing data set. A similar tendency was also observed in the external data set (Figures 5C and 5D). These results are consistent with those of the receiver operating characteristic analysis.

### Visualization of the Internal Features Learned by the Neural Networks

**Figure 6:**
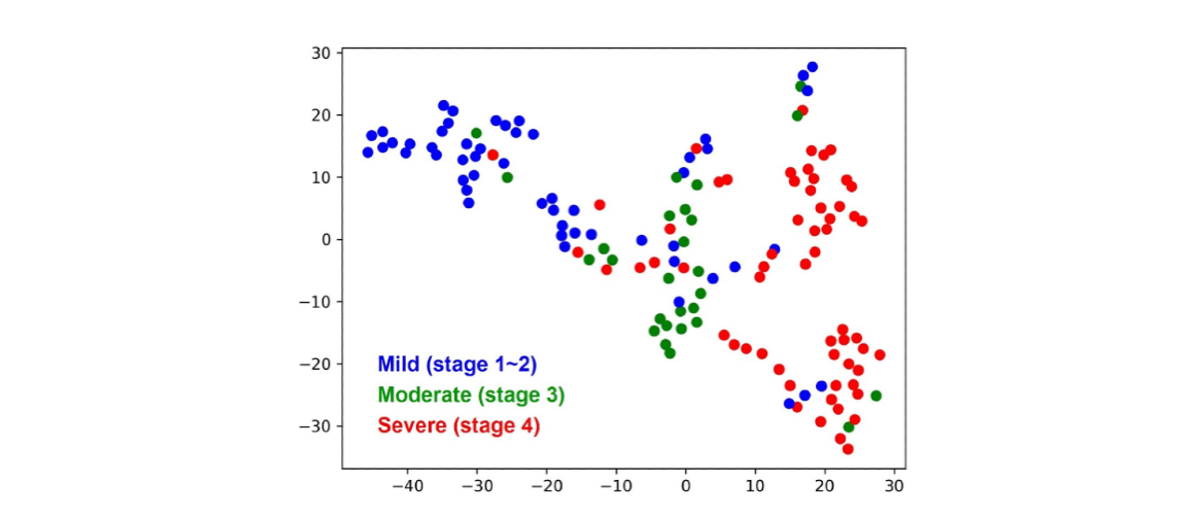
Visualization of the internal representations of the hybrid model for three severity classes by t-distributed stochastic neighbor embedding. Colored point clouds represented the different severity categories, showing how the neural network clustered the diseases.

The internal features learned by the hybrid model were examined in the internal testing data set by using the *t*-distributed stochastic neighbor embedding method [26]. Each point represented an input noncontrast CT image of a patient projected from the high-dimensional vector of the neural network’s last hidden layer into 2 dimensions. The mild (blue point cloud) and severe (red point cloud) groups showed clear clustering patterns and were split across the moderate group (green point cloud; Figure 6).

## Discussion

### Principal Findings and Comparison With Prior Work

Preoperative evaluation of tumor invasion depth determines individual treatment plans for GC. CT has been the first choice for preoperative GC evaluation and is important for clinical practice. However, routine preoperative determination is still not accurate enough, especially when radiologists carry out naked-eye evaluations. This study makes extensive use of the CT radiomics signature and the signature extracted by deep learning to generate a better prediction model to overcome the heterogeneity resulting from naked-eye evaluations. To our knowledge, this is the first study to demonstrate a CT-based model using radiomics and deep learning techniques for automated prediction of the T stage of GC. Our hybrid model demonstrated superior clinical utility to that of both the radiomics and deep learning models individually. Moreover, the hybrid model exhibited promising diagnostic performance in determining the pathological stage of GC.

Over the past decade, radiomics research has gained increasing attention, and a growing number of studies reported the use of radiomics in oncology [27]. At the very least, this could be an important complement to subjective evaluation by radiologists. Previous studies have illustrated that CT-based radiomics used alone or with deep learning or other statistical methods could predict the number of lymph node metastases [11,28], chemotherapy treatment responses, and overall survival [10,29] among patients with GC. Radiomics features are mathematically defined descriptors, while deep learning features are less intuitive due to the complexity of deep neural networks. The predictive values of the two are different and stackable [30]. A pilot study conducted by Sun et al [31] based on radiomics analysis reported that the model had a predictive AUC of 0.852 for the diagnostic T stage in rectal cancer. Yang et al [32] demonstrated that CT radiomics signatures exhibited favorable predictive performance for the T stage in esophageal carcinoma, with an AUC of 0.86. Furthermore, our study shows that the hybrid (83.7%) and deep learning (75.1%) models had higher accuracy than the radiomics model (69.8%) in evaluating the T stage based on 3 grades (mild, moderate, and severe groups) in the development, internal training, and external training data sets. In addition, the hybrid and deep learning models showed better performance than the radiomics model, with high agreement between the model’s prediction and ground truth. Thus, using the hybrid and deep learning models for pathological prediction is eligible and reliable. Furthermore, the hybrid model had higher accuracy than the deep learning model in the development data set (*P*<.001); however, no significant difference was observed in the internal (*P*=.25) or external (*P*=.15) testing data sets, which was possibly due to the limited sample sizes therein. By using the hybrid model, the diagnostic accuracy of the T stage could be improved, which can help clinicians make more precise treatment plans for patients with GC to avoid treatment delays or mistreatment. Furthermore, deep learning can reduce the workload of doctors and improve work efficiency.

Additionally, we aimed to better explore the predictive models constructed by the deep learning and hybrid models. The tumor invasion depth was evaluated separately in mild versus nonmild and nonsevere versus severe GCs. The radiomics, deep learning, and hybrid models displayed good performance in differentiating between mild and nonmild GC and severe and nonsevere GC in both the internal and external training data sets. Meanwhile, the hybrid model (AUC 0.972, 95% CI 0.897-0.997) outperformed both the radiomics (AUC 0.820, 95% CI 0.704-0.904; *P*=.002) and deep learning (AUC 0.818, 95% CI 0.703-0.903; *P*=.03) models in the external testing data set. Based on the hybrid model, for patients with stage T1 or T2 lesions, endoscopic resection or surgery was considered in combination with other examinations, while for patients with T3 or T4 lesions, adjuvant therapy was recommended. One retrospective study involving 572 patients with GC diagnosed at pathological stage T3 or T4 showed that a radiomics model based on CT images with deep learning is effective in discriminating serosa invasion in GC [33], which is consistent with our results. Patients with GC with stage T1~T3 disease have a lower risk of peritoneal metastasis than those with stage T4 disease, while in those with stage T4 disease, the hybrid model could assist clinicians in improving the detection accuracy of peritoneal metastasis, especially for regions where staging laparoscopy was not applicable widely. Furthermore, decision curve analysis revealed that the deep learning and hybrid models showed obviously higher net benefit than the radiomics model across the majority of the range of reasonable threshold probabilities, implying that the deep learning and hybrid models have certain clinical applicability. Furthermore, *t*-distributed stochastic neighbor embedding analysis revealed that different GC risk stratifications have their own distinct clusters. Based on the above results, the deep learning and hybrid models have good performance in distinguishing T stages in GC. Additionally, the hybrid model might perform better in distinguishing T stages than the deep learning and radiomics models.

### Clinical Implications

With respect to patient outcomes, the hybrid model can accurately predict the T stage directly from conventional CT images through automated processes. Furthermore, the hybrid model shows promise in aiding clinicians by offering a more dependable and accurate preoperative T staging diagnosis, which can influence real-world clinical decision-making. For example, by providing accurate T staging, neoadjuvant therapy can be arranged for patients with advanced GC. Through pretreatment evaluation of T stages, the hybrid model could help clinicians choose the correct treatment method. Following surgical procedures, we offer individualized treatment plans for patients through precise pathological diagnosis and selection of suitable adjuvant therapies.

### Ethical Implications

A prior investigation identified 3 key domains of ethical concerns related to the use of artificial intelligence (AI): algorithms, data, and practices [34]. These domains highlight the importance of obtaining informed consent and establishing data use agreements between data providers and third-party data aggregators. Furthermore, ensuring the quality of data used in AI algorithms, especially in the context of enhancing patient treatment decisions, is a significant area of concern. Furthermore, the integration of AI technology should not be viewed as a substitute for the collaborative decision-making process that is integral to optimal patient care. It is important to juxtapose these findings with the standard clinical practices currently used for T staging in GC.

### Limitations

There are several limitations in the study. First, this was a retrospective study that had a selection bias, and the limited sample size of images also contributed to a bias in model construction. Hence, for further validation and to enhance the generalizability of our results, a future prospective study with a large sample size is necessary. Second, our method requires manual segmentation of the tumor, which is time-consuming. Automated segmentation methods, as well as fully automated models, could be valuable in the future. Additionally, when tumor ROIs were outlined manually, there was some heterogeneity in the radiologists’ experience, and the radiologists needed to be calibrated. In addition, our study population included only Asian individuals; future studies from other centers are necessary to confirm the validity of this model prior to its potential clinical implementation. Furthermore, AI lacks the capacity to participate in complex dialogues with patients, as well as the ability to establish the necessary trust and empathy essential for fostering a therapeutic alliance that is crucial to the patient-physician relationship and to favorable treatment outcomes.

### Conclusions

The hybrid model can potentially distinguish T stages of GC more effectively than the deep learning or radiomics model and could be applied in clinical practice.

## Appendix

Multimedia Appendix 1 Supplementary tables and figures.

## Abbreviations

AI: artificial intelligence
AUC: area under the curve
CA19-9: carbohydrate antigen 19-9
CA72-4: serum cancer antigen 72-4
CA125: serum cancer antigen 125
CEA: carcinoembryonic antigen
CT: computed tomography
GC: gastric cancer
ICC: intraclass correlation coefficient
ROI: region of interest
TNM: tumor‐node‐metastasis
ViT: vision transformer

## Data Availability

The data and code are available upon reasonable request from the corresponding author.
