# Supplementary material for: Development and Validation of a Computed Tomography–Based Model for Noninvasive Prediction of the T Stage in Gastric Cancer: Multicenter Retrospective Study
Source: J Med Internet Res. 2024 Oct 9;26:e56851. doi: 10.2196/56851 (PMC11499715; doi:10.2196/56851)
Supplement: Multimedia Appendix 1 [file jmir_v26i1e56851_app1.pdf]

Supplementary Material

Section 1: Patient recruitment

This multicenter study design is shown in Figure S1. A total of 771 patients from three centers were enrolled; 148 were confirmed as T1 stage, 134 were confirmed as T2 stage, 146 were confirmed as T3 stage and 343 were confirmed as T4 stage. A total of 706 patients from West China Hospital between January 2013 and January 2021 met the inclusion/exclusion criteria. 65 patients from People's Hospital of Leshan and The First Affiliated Hospital of Chengdu Medical College in China between November 2017 and January 2021 met the inclusion/exclusion criteria.

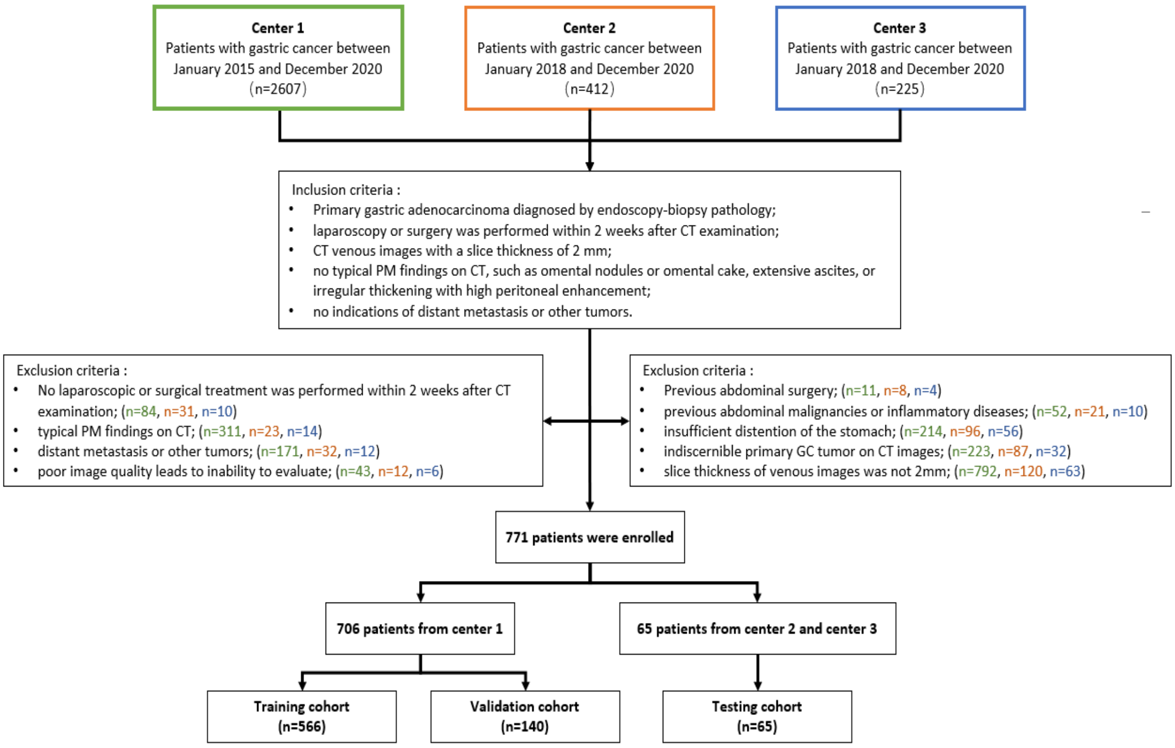

Figure S1. Flowchart of patient enrollment and study design.

Section 2: CT examinations

Prior to the CT examination, patients were requested to fast from food for at least 6 h and limit water intake to 600 – 1000 mL to achieve gastric distension prior to the examination. Patients were first trained to hold their breath during CT scanning. The scan covered the entire abdomen. The parameters of the CT protocol are listed in Table S1.

Table S1. The CT protocols of the three centers

Parameter	Center 1	Center 2	Center 3
**CT scanner**	Siemens Somatom Definition AS+, Siemens Somatom Definition	Siemens Somatom Definition AS +	Philips Brilliance 64
Tube voltage (KV)	120	120	120
Amperage (mAs)	210	210	110-170
Slice thickness (mm)	2	2	2
Slice interval (mm)	2	2	2
Field of view (cm)	35~50	35~50	35
Image matrix	512 × 512	512 × 512	512 × 512
rotation time (s)	0.5	\	0.5
Pitch	1.0	0.6	0.891
**Contrast agent**	Iopamiro, 370 mg I/mL	\	Ioversol, 300 mg I/mL
**Contrast agent infused Rate** (mL/s)	2.5 – 3.0	3.0	3.0
**Contrast agent infused dosage (**mL/kg**)**	1.2 – 1.5	1.2 – 1.5	1.2 – 1.5
**Portal vein phase CT images**	The precontrast phase, the arterial phase at the trigger, and the portal vein phase 30 s after the trigger were obtained with a trigger threshold of 170 HU in the aorta.	The precontrast phase, the arterial phase at the trigger, and the portal vein phase 30 s after the trigger were obtained with a trigger threshold of 170 HU in the aorta.	The precontrast phase, the arterial phase at the trigger, and the portal vein phase 30 s after the trigger were obtained with a trigger threshold of 170 HU in the aorta.

Table S2. The ICC values of the selected radiomics features.

Radiomics features	Intra-observer ICC	Inter-observer ICC
firstorder_Mean_wavelet-HHL	0.9875	0.9743
firstorder_RobustMeanAbsoluteDeviation_logarithm	0.8887	0.8535
firstorder_Skewness_squareroot	0.8652	0.8766
firstorder_Skewness_wavelet-LLH	0.9672	0.9491
firstorder_Skewness_wavelet-LLL	0.9683	0.9565
gldm_LowGrayLevelEmphasis_log-sigma-2-0-mm-3D	0.9789	0.9207
gldm_SmallDependenceLowGrayLevelEmphasis_wavelet-HLL	0.9340	0.9502
gldm_SmallDependenceLowGrayLevelEmphasis_wavelet-LHL	0.9112	0.9149
glrlm_ShortRunLowGrayLevelEmphasis_log-sigma-3-0-mm-3D	0.9498	0.9565
glszm_LargeAreaLowGrayLevelEmphasis_original	0.9933	0.9829
glszm_SizeZoneNonUniformity_squareroot	0.9624	0.9455
glszm_SmallAreaLowGrayLevelEmphasis_log-sigma-3-0-mm-3D	0.9483	0.9012
shape_MajorAxisLength_original	0.9167	0.9363
shape_Maximum2DDiameterRow_original	0.9541	0.8780
shape_Maximum2DDiameterSlice_original	0.9293	0.8921
